# *catena*-Poly[[bis­(4-carboxy­cyclo­hexane­carboxyl­ato-κ^2^ *O* ^1^,*O* ^1′^)cadmium(II)]-μ-1,4-bis­(imidazol-1-ylmeth­yl)benzene-κ^2^ *N* ^3^:*N* ^3′^]

**DOI:** 10.1107/S1600536809021618

**Published:** 2009-06-10

**Authors:** Bing-Bing Li, Bo Xiao

**Affiliations:** a School of Environmental Science and Engineering, Huazhong University of Science and Technology, Wuhan 430074, People’s Republic of China; b Department of Bioengineering, Henan University of Urban Construction, Pingdingshan 467000, People’s Republic of China

## Abstract

In the title coordination polymer, [Cd(C_8_H_11_O_4_)_2_(C_14_H_14_N_4_)]_*n*_, the Cd atom (site symmetry 2) is six-coordin­ated by two *O*,*O*′-bidentate 4-carboxy­cyclo­hexa­necarboxyl­ate (Hchdc) ligands and two N atoms from two different 1,4-bis­(imidazol-1-ylmeth­yl)benzene (1,4-bix) mol­ecules in a very distorted *cis*-CdN_2_O_4_ octa­hedral environment. The 1,4-bix mol­ecules act as bridging ligands that bind two Cd^II^ atoms, thus forming an infinite chain propagating in [100], which is decorated by the Hchdc anions. The structure is completed by O—H⋯O hydrogen bonds, which link the chains together.

## Related literature

For related structures, see: Qi *et al.* (2003 ▶). For background to coordination polymers, see: Chen & Liu (2002 ▶); Fang *et al.* (2006 ▶); Kim & Jung (2002 ▶); Lehn (1990 ▶); Batten & Robson (1998 ▶); Yang *et al.* (2008 ▶).
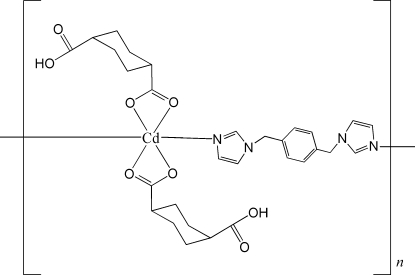


## Experimental

### 

#### Crystal data

[Cd(C_8_H_11_O_4_)_2_(C_14_H_14_N_4_)]
*M* *_r_* = 693.03
Orthorhombic, 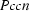

*a* = 12.6317 (5) Å
*b* = 19.9697 (12) Å
*c* = 12.3703 (7) Å
*V* = 3120.4 (3) Å^3^
*Z* = 4
Mo *K*α radiation
μ = 0.75 mm^−1^
*T* = 292 K
0.26 × 0.22 × 0.17 mm

#### Data collection

Oxford Diffraction Gemini R Ultra diffractometer
Absorption correction: multi-scan (*CrysAlis RED*; Oxford Diffraction, 2006 ▶) *T*_min_ = 0.816, *T*_max_ = 0.882
26676 measured reflections
3190 independent reflections
1658 reflections with *I* > 2σ(*I*)
*R*_int_ = 0.116

#### Refinement

*R*[*F* ^2^ > 2σ(*F* ^2^)] = 0.052
*wR*(*F* ^2^) = 0.120
*S* = 0.95
3190 reflections
195 parameters
H-atom parameters constrained
Δρ_max_ = 0.62 e Å^−3^
Δρ_min_ = −0.30 e Å^−3^

### 

Data collection: *CrysAlis CCD* (Oxford Diffraction, 2006 ▶); cell refinement: *CrysAlis CCD*; data reduction: *CrysAlis RED* (Oxford Diffraction, 2006 ▶); program(s) used to solve structure: *SHELXS97* (Sheldrick, 2008 ▶); program(s) used to refine structure: *SHELXL97* (Sheldrick, 2008 ▶); molecular graphics: *SHELXTL-Plus* (Sheldrick, 2008 ▶); software used to prepare material for publication: *SHELXL97*.

## Supplementary Material

Crystal structure: contains datablocks global, I. DOI: 10.1107/S1600536809021618/hb2986sup1.cif

Structure factors: contains datablocks I. DOI: 10.1107/S1600536809021618/hb2986Isup2.hkl

Additional supplementary materials:  crystallographic information; 3D view; checkCIF report

## Figures and Tables

**Table:** 

Cd1—N1	2.249 (4)
Cd1—O1	2.306 (4)
Cd1—O2	2.384 (4)

**Table:** 

O1—Cd1—O2	55.00 (14)

**Table 2:** Hydrogen-bond geometry (Å, °)

*D*—H⋯*A*	*D*—H	H⋯*A*	*D*⋯*A*	*D*—H⋯*A*
O4—H4⋯O2^i^	0.82	1.86	2.644 (6)	161

Symmetry code: (i) 
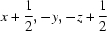
.

**Fig. 1.**
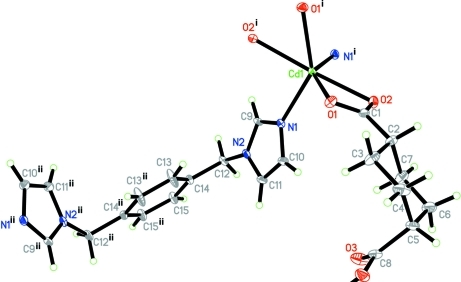
The structure of (I), showing displacement ellipsoids drawn at the 20% probability level. Symmetry code: (i) 3/2 - x, 1/2 - y, z; (ii) 5/2 - x, -1/2 - y, z.

**Fig. 2.**
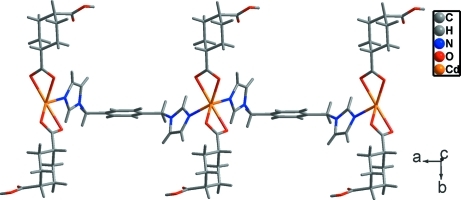
View of the chain structure of (I).

## supplementary crystallographic information

### Comment

The rational design and synthesis of metal-organic coordination polymers have received intense interest due to their fascinating structural topologies and potential applications as functional materials (e.g. Fang *et al.*, 2006). These coordination polymers can be specially designed by the careful selection of metal cations with preferred coordination geometries, the nature of the anions, the structure of the connecting ligands, and the reaction conditions (Kim & Jung, 2002). The selection of ligand is extremely important because changing the structures of the ligands can control and adjust the topologies of coordination frameworks. Among these mentioned above, chain structures have received much attention in coordination chemistry and life sciecnce (Lehn, 1990). So far, many chain complexes have been generated by self-assembly processes (Chen & Liu, 2002). In this regard, metal 1,4-benzenedicarboxylates (1,4-bdc) have been widely studied (Qi *et al.*, 2003). However, so far, less attention has been given to the 1,4-cyclohexanedicarboxylic acid ligand (H_2_chdc). The H_2_chdc as an important analogues of 1,4-bdc may be a good candidate for the construction of metal-organic architectures. On the other hand, 4,4'-byridine is a rigid rod-like spacer, well known in the construction of metal-organic polymers, and it has adopted numerous interesting supramolecular architectures (Batten & Robson, 1998). However, flexible ligands such as 1,4-bis(imidazole-1-ylmethyl)-benzene (1,4-bix) have not been so well explored to date (Yang *et al.*, 2008). In this work, the combination of 1,4-bix with H_2_chdc and Cd^II^ cations resulted in the title compound [Cd(1,4-bix)(Hchdc)_2_], (I), a new one-dimensional chain coordination polymer.

The selected bond lengthes and angles are listed in Table 1. In compound (I), the Cd^II^ atom is is six-coordinated by four carboxylate O atoms from two different Hchdc ligands, and two N atoms from two different 1,4-bix molecules in a distorted octahedral environment (Fig. 1). The O1, O2, O2^i^ and N1^i^ atoms comprise the basal plane, whereas the N1 and O1^i^ occupy the axial positions of the octahedron. As shown in Fig. 2, each 1,4-bix acts as a bridging ligand that binds two Cd^II^ atoms, thus forming a unique chain. The chain is decorated with Hchdc molecules alternately at two sides. Furthermore, the O—H···O hydrogen bonds link the chains together, stablizing the structure of (I).

### Experimental

A mixture of CdCl_2_^.^2H_2_O (0.5 mmol), H_2_chdc acid (0.5 mmol), 1,4-bix (0.5 mmol), and H_2_O (500 mmol) was adjusted to pH = 5.8 by addition of aqueous NaOH solution, and heated at 453 K for 5 days. After the mixture was slowly cooled to room temperature, colorless blocks of (I) were recovered in a 28% yield.

### Refinement

The H atoms were positioned geometrically (C—H = 0.93–0.97 Å, O—H = 0.82Å) and refined as riding, with *U*_iso_(H)=1.2*U*_eq_(carrier).

### Crystal data

**Table:**

[Cd(C_8_H_11_O_4_)_2_(C_14_H_14_N_4_)]	*F*(000) = 1424
*M**_r_* = 693.03	*D*_x_ = 1.475 Mg m^−^^3^
Orthorhombic, *P**c**c**n*	Mo *K*α radiation, λ = 0.71073 Å
Hall symbol: -P 2ab 2ac	Cell parameters from 3190 reflections
*a* = 12.6317 (5) Å	θ = 3.0–26.5°
*b* = 19.9697 (12) Å	µ = 0.75 mm^−^^1^
*c* = 12.3703 (7) Å	*T* = 292 K
*V* = 3120.4 (3) Å^3^	Block, colorless
*Z* = 4	0.26 × 0.22 × 0.17 mm

### Data collection

**Table:**

Oxford Diffraction Gemini R Ultra diffractometer	3190 independent reflections
Radiation source: fine-focus sealed tube	1658 reflections with *I* > 2σ(*I*)
graphite	*R*_int_ = 0.116
Detector resolution: 10.0 pixels mm^-1^	θ_max_ = 26.5°, θ_min_ = 4.7°
ω scans	*h* = −15→15
Absorption correction: multi-scan (*CrysAlis RED*; Oxford Diffraction, 2006)	*k* = −24→24
*T*_min_ = 0.816, *T*_max_ = 0.882	*l* = −15→15
26676 measured reflections	

### Refinement

**Table:**

Refinement on *F*^2^	Primary atom site location: structure-invariant direct methods
Least-squares matrix: full	Secondary atom site location: difference Fourier map
*R*[*F*^2^ > 2σ(*F*^2^)] = 0.052	Hydrogen site location: inferred from neighbouring sites
*wR*(*F*^2^) = 0.120	H-atom parameters constrained
*S* = 0.95	*w* = 1/[σ^2^(*F*_o_^2^) + (0.0575*P*)^2^] where *P* = (*F*_o_^2^ + 2*F*_c_^2^)/3
3190 reflections	(Δ/σ)_max_ < 0.001
195 parameters	Δρ_max_ = 0.62 e Å^−^^3^
0 restraints	Δρ_min_ = −0.30 e Å^−^^3^

### Special details

**Table:**

Geometry. All e.s.d.'s (except the e.s.d. in the dihedral angle between two l.s. planes) are estimated using the full covariance matrix. The cell e.s.d.'s are taken into account individually in the estimation of e.s.d.'s in distances, angles and torsion angles; correlations between e.s.d.'s in cell parameters are only used when they are defined by crystal symmetry. An approximate (isotropic) treatment of cell e.s.d.'s is used for estimating e.s.d.'s involving l.s. planes.
Refinement. Refinement of *F*^2^ against ALL reflections. The weighted *R*-factor *wR* and goodness of fit *S* are based on *F*^2^, conventional *R*-factors *R* are based on *F*, with *F* set to zero for negative *F*^2^. The threshold expression of *F*^2^ > σ(*F*^2^) is used only for calculating *R*-factors(gt) *etc*. and is not relevant to the choice of reflections for refinement. *R*-factors based on *F*^2^ are statistically about twice as large as those based on *F*, and *R*- factors based on ALL data will be even larger.

### Fractional atomic coordinates and isotropic or equivalent isotropic displacement parameters (Å^2^)

**Table:**

	*x*	*y*	*z*	*U*_iso_*/*U*_eq_	
C1	0.7200 (5)	0.1255 (3)	0.1946 (5)	0.0556 (16)	
C2	0.7134 (5)	0.0551 (3)	0.1562 (5)	0.0703 (18)	
H2	0.6472	0.0495	0.1158	0.084*	
C3	0.8075 (6)	0.0347 (3)	0.0813 (6)	0.089 (2)	
H3A	0.8072	0.0627	0.0172	0.107*	
H3B	0.8738	0.0420	0.1190	0.107*	
C4	0.7998 (7)	−0.0363 (4)	0.0488 (6)	0.106 (3)	
H4A	0.8606	−0.0478	0.0045	0.127*	
H4B	0.7367	−0.0427	0.0054	0.127*	
C5	0.7956 (5)	−0.0833 (3)	0.1467 (6)	0.082 (2)	
H5	0.7788	−0.1281	0.1192	0.099*	
C6	0.7065 (7)	−0.0639 (4)	0.2243 (7)	0.098 (2)	
H6A	0.6387	−0.0729	0.1905	0.118*	
H6B	0.7113	−0.0910	0.2891	0.118*	
C7	0.7123 (6)	0.0065 (3)	0.2537 (6)	0.089 (2)	
H7A	0.7758	0.0137	0.2961	0.107*	
H7B	0.6521	0.0172	0.2992	0.107*	
C8	0.9032 (6)	−0.0881 (3)	0.2022 (7)	0.078 (2)	
C9	0.9060 (3)	0.2554 (3)	0.4765 (4)	0.0524 (13)	
H9	0.8851	0.2997	0.4856	0.063*	
C10	0.9221 (4)	0.1567 (3)	0.4138 (5)	0.0657 (16)	
H10	0.9152	0.1192	0.3699	0.079*	
C11	0.9818 (5)	0.1601 (3)	0.5038 (5)	0.0665 (18)	
H11	1.0218	0.1258	0.5339	0.080*	
C12	1.0206 (3)	0.2510 (3)	0.6389 (4)	0.0612 (14)	
H12A	0.9962	0.2257	0.7010	0.073*	
H12B	0.9972	0.2969	0.6481	0.073*	
C13	1.1951 (4)	0.2506 (5)	0.5441 (5)	0.125 (4)	
H13	1.1592	0.2521	0.4785	0.150*	
C14	1.1390 (3)	0.2497 (3)	0.6368 (4)	0.0479 (11)	
C15	1.1954 (4)	0.2499 (4)	0.7277 (5)	0.0693 (15)	
H15	1.1599	0.2498	0.7936	0.083*	
N1	0.8735 (3)	0.2166 (2)	0.3967 (4)	0.0527 (11)	
N2	0.9721 (3)	0.2237 (2)	0.5420 (4)	0.0515 (12)	
O1	0.7903 (3)	0.1640 (2)	0.1601 (3)	0.0683 (11)	
O2	0.6563 (3)	0.1466 (2)	0.2657 (4)	0.0700 (12)	
O3	0.9194 (5)	−0.0730 (3)	0.2950 (5)	0.120 (2)	
O4	0.9764 (4)	−0.1119 (2)	0.1406 (4)	0.0951 (15)	
H4	1.0323	−0.1134	0.1741	0.143*	
Cd1	0.7500	0.2500	0.27740 (4)	0.04487 (19)	

### Atomic displacement parameters (Å^2^)

**Table:**

	*U*^11^	*U*^22^	*U*^33^	*U*^12^	*U*^13^	*U*^23^
C1	0.062 (4)	0.045 (3)	0.059 (4)	0.007 (3)	−0.007 (3)	0.005 (3)
C2	0.096 (5)	0.045 (3)	0.069 (4)	0.000 (3)	0.000 (4)	0.002 (3)
C3	0.149 (6)	0.058 (4)	0.061 (4)	−0.006 (4)	0.036 (5)	0.000 (4)
C4	0.118 (6)	0.103 (6)	0.096 (6)	0.048 (5)	−0.013 (5)	−0.008 (5)
C5	0.085 (4)	0.068 (4)	0.093 (6)	0.022 (4)	−0.020 (4)	0.014 (4)
C6	0.095 (5)	0.078 (5)	0.123 (7)	0.020 (4)	0.014 (5)	0.016 (5)
C7	0.111 (6)	0.065 (4)	0.092 (6)	−0.013 (4)	0.027 (4)	0.005 (4)
C8	0.089 (5)	0.058 (4)	0.087 (6)	0.029 (4)	−0.001 (4)	0.002 (4)
C9	0.036 (2)	0.060 (3)	0.062 (3)	0.015 (3)	−0.002 (3)	0.001 (4)
C10	0.067 (4)	0.055 (4)	0.075 (5)	0.009 (3)	−0.018 (3)	0.002 (3)
C11	0.060 (4)	0.062 (4)	0.077 (5)	0.014 (3)	−0.018 (3)	0.006 (3)
C12	0.037 (2)	0.094 (4)	0.052 (3)	0.009 (4)	−0.006 (2)	0.000 (4)
C13	0.042 (3)	0.291 (12)	0.042 (4)	−0.020 (7)	−0.008 (3)	−0.003 (6)
C14	0.040 (2)	0.062 (3)	0.042 (3)	−0.002 (3)	0.001 (2)	−0.009 (4)
C15	0.048 (3)	0.119 (5)	0.041 (3)	−0.006 (5)	0.004 (2)	−0.004 (5)
N1	0.039 (2)	0.056 (3)	0.064 (3)	0.000 (2)	−0.007 (2)	0.006 (3)
N2	0.030 (2)	0.069 (3)	0.056 (3)	−0.0009 (19)	−0.002 (2)	0.006 (2)
O1	0.083 (3)	0.058 (2)	0.064 (3)	0.001 (2)	0.021 (2)	−0.004 (2)
O2	0.051 (2)	0.059 (2)	0.100 (3)	−0.0010 (18)	0.015 (2)	−0.025 (2)
O3	0.143 (5)	0.130 (5)	0.088 (4)	0.059 (4)	−0.019 (4)	−0.005 (4)
O4	0.077 (3)	0.099 (4)	0.110 (4)	0.027 (3)	−0.007 (3)	−0.027 (3)
Cd1	0.0318 (2)	0.0523 (3)	0.0505 (3)	0.0036 (3)	0.000	0.000

### Geometric parameters (Å, °)

**Table:**

C1—O1	1.250 (6)	C9—N2	1.325 (7)
C1—O2	1.265 (6)	C9—H9	0.9300
C1—C2	1.487 (8)	C10—C11	1.347 (8)
C1—Cd1	2.716 (6)	C10—N1	1.361 (6)
C2—C7	1.548 (8)	C10—H10	0.9300
C2—C3	1.561 (8)	C11—N2	1.360 (7)
C2—H2	0.9800	C11—H11	0.9300
C3—C4	1.478 (9)	C12—N2	1.453 (7)
C3—H3A	0.9700	C12—C14	1.496 (6)
C3—H3B	0.9700	C12—H12A	0.9700
C4—C5	1.533 (10)	C12—H12B	0.9700
C4—H4A	0.9700	C13—C14	1.347 (7)
C4—H4B	0.9700	C13—C13^i^	1.388 (11)
C5—C8	1.526 (9)	C13—H13	0.9300
C5—C6	1.529 (9)	C14—C15	1.331 (7)
C5—H5	0.9800	C15—C15^i^	1.379 (10)
C6—C7	1.453 (9)	C15—H15	0.9300
C6—H6A	0.9700	O4—H4	0.8200
C6—H6B	0.9700	Cd1—N1	2.249 (4)
C7—H7A	0.9700	Cd1—O1	2.306 (4)
C7—H7B	0.9700	Cd1—O2	2.384 (4)
C8—O3	1.205 (8)	Cd1—N1^ii^	2.249 (4)
C8—O4	1.288 (8)	Cd1—O1^ii^	2.306 (4)
C9—N1	1.320 (7)	Cd1—O2^ii^	2.384 (4)
			
O1—C1—O2	119.0 (5)	C11—C10—N1	109.7 (5)
O1—C1—C2	120.8 (5)	C11—C10—H10	125.2
O2—C1—C2	120.2 (6)	N1—C10—H10	125.2
C1—C2—C7	110.2 (5)	C10—C11—N2	106.5 (5)
C1—C2—C3	113.2 (5)	C10—C11—H11	126.8
C7—C2—C3	107.8 (5)	N2—C11—H11	126.8
C1—C2—H2	108.5	N2—C12—C14	113.6 (4)
C7—C2—H2	108.5	N2—C12—H12A	108.8
C3—C2—H2	108.5	C14—C12—H12A	108.8
C4—C3—C2	111.2 (6)	N2—C12—H12B	108.8
C4—C3—H3A	109.4	C14—C12—H12B	108.8
C2—C3—H3A	109.4	H12A—C12—H12B	107.7
C4—C3—H3B	109.4	C14—C13—C13^i^	121.7 (3)
C2—C3—H3B	109.4	C14—C13—H13	119.2
H3A—C3—H3B	108.0	C13^i^—C13—H13	119.2
C3—C4—C5	112.0 (6)	C15—C14—C13	115.9 (4)
C3—C4—H4A	109.2	C15—C14—C12	121.3 (4)
C5—C4—H4A	109.2	C13—C14—C12	122.7 (5)
C3—C4—H4B	109.2	C14—C15—C15^i^	122.4 (3)
C5—C4—H4B	109.2	C14—C15—H15	118.8
H4A—C4—H4B	107.9	C15^i^—C15—H15	118.8
C8—C5—C6	112.9 (6)	C9—N1—C10	105.1 (5)
C8—C5—C4	111.2 (6)	C9—N1—Cd1	122.2 (4)
C6—C5—C4	111.5 (6)	C10—N1—Cd1	132.5 (4)
C8—C5—H5	106.9	C9—N2—C11	106.9 (5)
C6—C5—H5	106.9	C9—N2—C12	126.2 (5)
C4—C5—H5	106.9	C11—N2—C12	126.8 (5)
C7—C6—C5	111.5 (6)	C1—O1—Cd1	94.9 (3)
C7—C6—H6A	109.3	C1—O2—Cd1	90.9 (3)
C5—C6—H6A	109.3	C8—O4—H4	109.5
C7—C6—H6B	109.3	N1—Cd1—N1^ii^	97.9 (2)
C5—C6—H6B	109.3	N1—Cd1—O1	92.24 (16)
H6A—C6—H6B	108.0	N1^ii^—Cd1—O1	141.92 (15)
C6—C7—C2	114.3 (6)	N1—Cd1—O1^ii^	141.92 (15)
C6—C7—H7A	108.7	N1^ii^—Cd1—O1^ii^	92.24 (16)
C2—C7—H7A	108.7	O1—Cd1—O1^ii^	102.0 (2)
C6—C7—H7B	108.7	N1—Cd1—O2	97.33 (15)
C2—C7—H7B	108.7	N1^ii^—Cd1—O2	87.24 (15)
H7A—C7—H7B	107.6	O1—Cd1—O2	55.00 (14)
O3—C8—O4	122.2 (7)	O1^ii^—Cd1—O2	119.83 (15)
O3—C8—C5	124.4 (7)	N1—Cd1—O2^ii^	87.24 (15)
O4—C8—C5	113.3 (7)	N1^ii^—Cd1—O2^ii^	97.33 (15)
N1—C9—N2	111.8 (6)	O1—Cd1—O2^ii^	119.83 (15)
N1—C9—H9	124.1	O1^ii^—Cd1—O2^ii^	55.00 (14)
N2—C9—H9	124.1	O2—Cd1—O2^ii^	173.1 (2)

Symmetry codes: (i) −*x*+5/2, −*y*+1/2, *z*; (ii) −*x*+3/2, −*y*+1/2, *z*.

### Hydrogen-bond geometry (Å, °)

**Table:**

*D*—H···*A*	*D*—H	H···*A*	*D*···*A*	*D*—H···*A*
O4—H4···O2^iii^	0.82	1.86	2.644 (6)	161

Symmetry codes: (iii) *x*+1/2, −*y*, −*z*+1/2.

## References

[bb1] Batten, S. R. & Robson, R. (1998). *Angew. Chem. Int. Ed.* **37**, 1460–1494. 10.1002/(SICI)1521-3773(19980619)37:11<1460::AID-ANIE1460>3.0.CO;2-Z 29710936

[bb2] Chen, X. M. & Liu, G. F. (2002). *Chem. Eur. J.* **8**, 4811–4817. 10.1002/1521-3765(20021018)8:20<4811::AID-CHEM4811>3.0.CO;2-R 12561122

[bb3] Fang, Q.-R., Zhu, G.-S., Xue, M., Zhang, Q.-L., Sun, J.-Y., Guo, X.-D., Qiu, S.-L., Xu, S.-T., Wang, P., Wang, D.-J., Wei, Y. (2006). *Chem. Eur. J.* **12**, 3754-3758. 10.1002/chem.200500963 16514680

[bb4] Kim, Y. J. & Jung, D.-Y. (2002). *Chem. Commun.* pp. 908–909. 10.1039/b200658h 12123039

[bb5] Lehn, J. M. (1990). *Angew. Chem., Int. Ed. Engl.* **29**, 1304–1305.

[bb6] Oxford Diffraction (2006). *CrysAlis CCD* and *CrysAlis RED* Oxford Diffraction Ltd, Abingdon, England.

[bb7] Qi, Y., Wang, Y., Hu, C., Cao, M., Mao, L. & Wang, E. (2003). *Inorg. Chem.* **42**, 8519–8523. 10.1021/ic034796p 14658908

[bb8] Sheldrick, G. M. (2008). *Acta Cryst.* A**64**, 112–122. 10.1107/S0108767307043930 18156677

[bb9] Yang, J., Ma, J.-F., Batten, S. R. & Su, Z.-M. (2008). *Chem. Commun.* pp. 2233–2235. 10.1039/b800199e 18463750

